# Cardiac Hemangioma of RVOT in a Patient with Atypical Chest Pain

**DOI:** 10.1155/2014/285479

**Published:** 2014-07-08

**Authors:** Hossein Vakili, Isa Khaheshi, Mahnoosh Foroughi, Hamid Ghaderi, Shooka Esmaeeli, Mostafa Jafari

**Affiliations:** ^1^Cardiovascular Research Center, Modarres Hospital, Shahid Beheshti University of Medical Sciences, Saadat Abad, Kaj Square, Tehran 22074088, Iran; ^2^Cardiovascular Research Center and, Department of Cardiovascular Surgery, Modarres Hospital, Shahid Beheshti University of Medical Sciences, Tehran 4739, Iran; ^3^Student Scientific Research Center (SSRC), Tehran University of Medical Sciences (TUMS), Tehran 14155-6537, Iran; ^4^Department of Pathology, Modarres Hospital, Shahid Beheshti University of Medical Sciences, Tehran 19857-17443, Iran

## Abstract

A 40-year-old man presented with atypical chest pain and fatigue from 15 days ago a suspicious mass in the right ventricle based on a bed side transthoracic echocardiography. Preoperative diagnosis of a cardiac hemangioma comes to mind in a minority of cases. In our case, a cardiac tumor was diagnosed and the vascular nature of the tumor was suggested by vascular blush on the coronary angiography. In addition, right ventriculotomy was the approach of choice in our case because of its inaccessibility and its particular location.

## 1. Introduction

Cardiac primary tumors are not common and they are often diagnosed postmortem because they are frequently asymptomatic. The incidence of primary cardiac tumors distinguished at autopsy is about 0.02%. Three quarters of cardiac tumors can be categorized a benign tumor on histology. Myxoma is the most common tumor among cardiac tumors [1, 2].

Hemangioma generally arises from the gastrointestinal tract or the cutaneous structures and is an extremely rare heart tumor. Capillary hemangioma is one of the three histological types of cardiac hemangioma, including capillary type, cavernous type, and mixed [3, 4].

## 2. Case Presentation

A 40-year-old man was referred to our center with atypical chest pain and fatigue from 15 days ago and a suspicious mass in the right ventricle based on a bed side transthoracic echocardiography in another hospital.

He was a cigarette smoker (20 packs/year) for about 16 years and he had no other important point in his past history.

On physical examinations, blood pressure was 110/65 mmHg, heart rate was 72/min, respiratory rate was 14/min, and body temperature was 37°C. Other physical examinations were unremarkable.

Hematology, biochemistry, and coagulation tests and cardiac enzymes were all in normal ranges.

**Figure 1:**
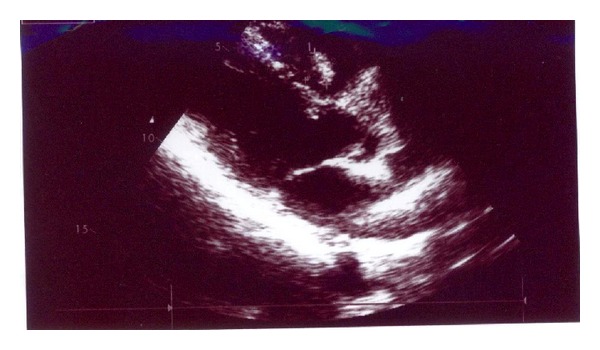
Transthoracic echocardiography revealed a round pedunculated semimobile dense mass (1.6 cm ∗ 1.8 cm) in right ventricular outflow tract (RVOT) with attachment to the base of the septum.

**Figure 2:**
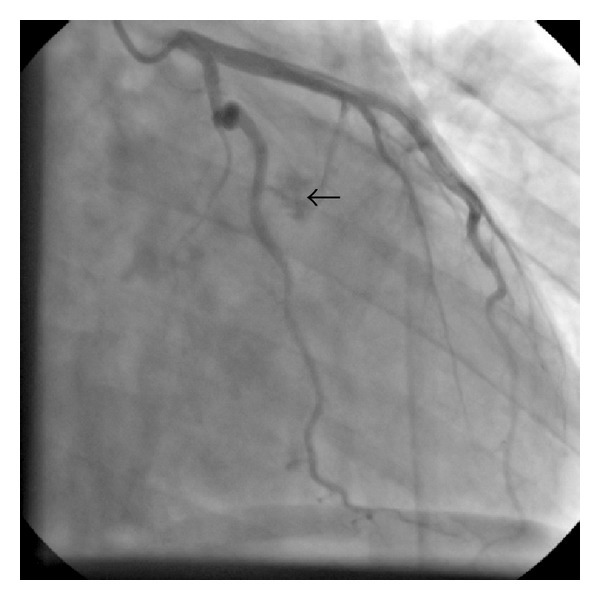
Right anterior caudal view of left coronary angiography showed delayed vascular blush (arrow) indicating a vascular mass which was fed from septal branches of left anterior descending artery.

His electrocardiogram (ECG) showed normal sinus rhythm, normal axis, and no ST segment or T wave changes. A comprehensive transthoracic echocardiography revealed ejection fraction (EF) = 60%, mild mitral regurgitation, trivial tricuspid regurgitation, systolic pulmonary artery pressure (SPAP) = 30 MMHG, and a round pedunculated semimobile dense mass (1.6 cm ∗ 1.8 cm) in right ventricular outflow tract (RVOT) with attachment to the base of the septum with no obstruction of RVOT (Figure 1). Coronary angiography was performed to exclude obstructive coronary artery disease before cardiac surgery which showed normal coronary arteries and delayed vascular blush indicating a vascular mass which was fed from septal branches of left anterior descending artery (Figure 2).

**Figure 3:**
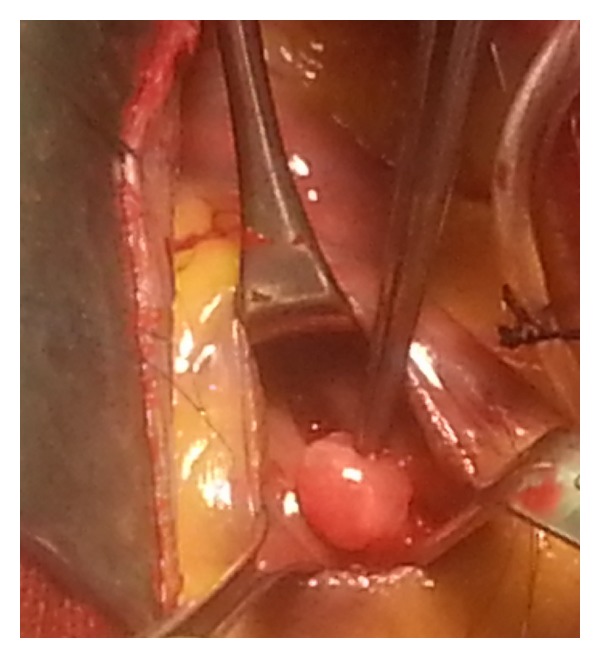
A 1.5 × 1.5 cm polypoid mass was found in right ventriculotomy approach.

The patient was candidate for elective excision of the cardiac mass. The right atrium was open but due to the mass location and its inaccessibility, it was approached by right ventriculotomy. A 1.5 cm × l.5 cm polypoid mass was found and excised with a 2 mm margin (Figure 3).

**Figure 4:**
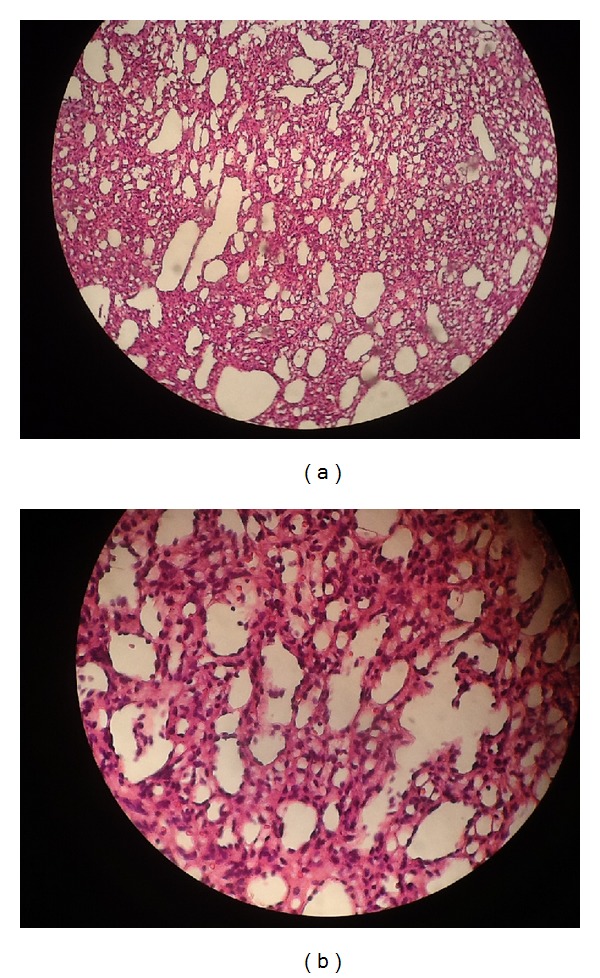
(a) and (b) showed vascular lesion containing small vessels of capillary caliber; closely packed spindle cells are also seen with neoformed spaces that contain little blood.

The specimen consisted of an ovaloid grayish-creamy elastic tissue. Microscopic findings showed vascular lesion containing small vessels of capillary caliber; closely packed spindle cells were also seen with neoformed spaces that contain little blood, compatible with capillary hemangioma (Figure 4). The patient was discharged on the 6th postoperative day with good general condition.

## 3. Discussion

Dyspnea, arrhythmias, atypical chest pain, pericardial effusion, congestive heart failure, ventricular outflow tract obstruction, embolic events, or sudden cardiac death may be features of the cardiac hemangioma; however, it is commonly asymptomatic [5].

Echocardiography is the choice diagnostic imaging modality to suitably screen for cardiac tumors including cardiac hemangiomas [3, 4]. Computed tomography scan and magnetic resonance imaging are complementary methods in the diagnostic workup of cardiac tumors for assessment of the scope of myocardial and local invasion. Coronary angiography will typically be an essential part of the preoperative evaluation to exclude obstructive coronary artery disease and to appraise the feeding arteries or disclose encasement of coronary vessels by the tumor. The natural history of cardiac hemangioma is changeable. It can continue to proliferate, die down to grow, or revert [3, 6, 7]. Complete tumor excision is suggested for tissue diagnosis and impending cure, with a likely good long-term prognosis. The rate of recurrence after surgical resection is indefinite [8]. A variety of surgical approaches have been suggested. Median sternotomy with cardiopulmonary bypass is generally necessary for intracardiac tumors. Whereas right-sided tumors can be approached by a right atriotomy, a left ventriculotomy for LV tumors is the best way to avoid postoperative LV dysfunction and arrhythmia [9].

This case underscores the importance of the echocardiography as an acceptable imaging modality for detecting cardiac tumors including cardiac hemangioma. Preoperative diagnosis of a cardiac hemangioma, however, comes to mind in a minority of cases. In our case, a cardiac tumor was diagnosed and the vascular nature of the tumor was supposed by vascular blush on the coronary angiography. In addition, right ventriculotomy was the approach of choice in our case because of its inaccessibility and its particular location.
